# Primary Nasopharyngeal Tuberculosis: A Diagnostic Challenge

**DOI:** 10.22038/IJORL.2022.64781.3218

**Published:** 2023-01

**Authors:** Ahmad-Hazim-Hazlami Ahmad Nizar, Ramiza-Ramza Ramli, Mohd-Najeb Soleh, Ikmal-Hisyam Bakrin

**Affiliations:** 1 *Department of Otorhinolaryngology - Head & Neck Surgery, School of Medical Sciences, University Sains Malaysia Health Campus, 16150 Kubang Kerian, Kelantan, Malaysia.*; 2 *Department of Otorhinolaryngology - Head & Neck Surgery, Pantai Hospital Sungai Petani, 08000 Sungai Petani, Kedah, Malaysia.*; 3 *Department of Pathology, Faculty of Medicine and Health Sciences, University Putra Malaysia, 43000 Serdang, Selangor, Malaysia.*

**Keywords:** Lymphadenopathy, Nasopharyngeal mass, Neck mass, Tuberculosis

## Abstract

**Introduction:**

Primary nasopharyngeal tuberculosis (NPTB) is a rare disease but should not be missed as one of the differential diagnoses for cervical lymphadenopathy or nasopharyngeal mass.

**Case Report:**

We describe a case of a 38 year old lady, who presented with bilateral cervical lymphadenopathy associated with intermittent fever. Nasoendoscopy examination and computed tomography scan of the neck revealed a centrally located mass predominantly at the left posterior nasopharyngeal wall without obliteration of both fossae of Rosenmuller. Typical histopathological features of necrotizing granulomatous lymphadenitis together with the common clinical presentation of cervical lymphadenopathy and nasoendoscopy findings of nasopharyngeal mass conclude the diagnosis of nasopharyngeal tuberculosis. With anti-tuberculous therapy, the cervical lymphadenopathy and nasopharyngeal mass were completely resolved.

**Conclusion:**

Nasopharyngeal tuberculosis is an uncommon disease with great diagnostic challenges and with early diagnosis and adequate treatment, NPTB carries a good prognosis with complete disease resolution.

## Introduction

Tuberculosis (TB) is known to be one of the oldest infections in the history of humankind, which has caused significant morbidity and mortality since it was first recorded. According to the World Health Organization (WHO) Tuberculosis Report 2020, Mycobacterium tuberculosis had blighted about 10 million people worldwide in 2019 (1). Asia remained one of the regions with the highest TB burden in the world. Globally, the mortality from TB infection among both HIV-negative and positive individuals had shown a declining trend which reflects the continuous effort made to improve the prevention, detection and management of the disease (1). TB can be classified into pulmonary and extrapulmonary, in which the head and neck TB occurrence is only about 10%. The most common affected site in extrapulmonary TB is the cervical lymph nodes, pleura, bone and joints, urogenital tract, and meninges. Other locations including the nasopharyngeal area, are less commonly affected but shouldn’t be disregarded in the differential diagnosis(2). 

## Case Report

38 years old Malay lady presented with a month’s history of bilateral neck swelling. The swelling was painless, gradually increasing in size and did not reduce any neck movement. She also experienced intermittent fever but was not associated with any other constitutional symptoms such as chills, weight loss, anorexia and night sweats. There was no history of epistaxis, rhinorrhea, nasal blockage, chronic cough, sore throat, hoarseness, ear fullness, tinnitus, or hearing impairment. 

She sought treatment at the health clinic and was prescribed with a course of oral augmentin. However, the neck swelling persisted. She had no history of contact with pulmonary tuberculosis-infected individuals and no history of malignancy in her family.

Neck examination revealed two lymph nodes over the left posterior triangle at level 5a, each measuring 1.5cm x 1.5cm and 3cm x 3cm, firm in consistency and non-tender.

**Fig 1:**
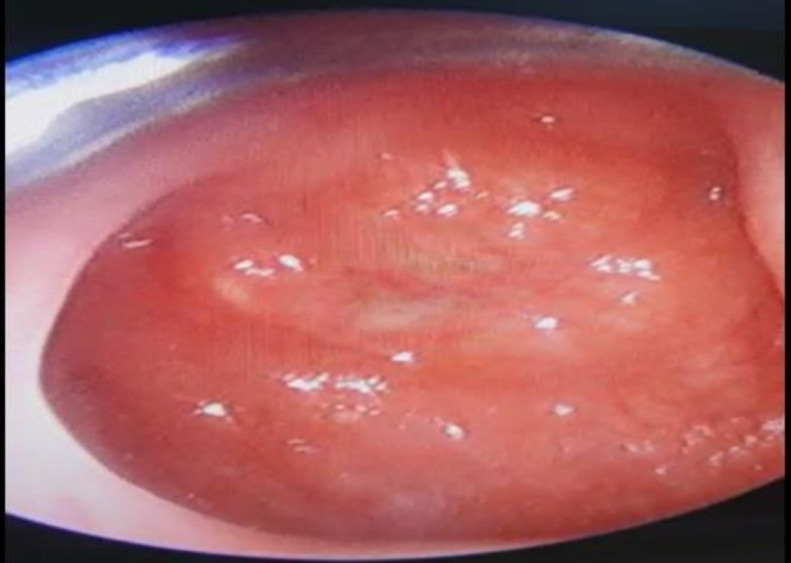
Centrally located mass predominantly at the left posterior nasopharyngeal wall with an irregular surface

On the right side of the neck, there was one lymph node at level 2 measuring 3cm x 3cm with similar characteristics. Oropharynx and ear examinations were unremarkable. Nasoendoscopy examination revealed a centrally located mass predominantly at the left posterior nasopharyngeal wall with an irregular surface but an otherwise normal fossa of Rosenmuller (FOR) and Eustachian tube (Figure 1). 

Blood parameters were within normal limits with the erythrocyte sedimentation rate (ESR) value of 14 mm/hour and a negative sputum acid-fast bacilli (AFB) staining. Chest radiography showed normal lung findings. 

Fine needle aspiration (FNAC) was performed over the left posterior triangle lymph node and showed features consistent with the necrotic lymph node. However, tuberculous lymphadenitis or tumour necrosis cannot be excluded from the cytological findings. Therefore, a contrasted enhanced computed tomography (CECT) scan was done and revealed a well-defined enhancing hypodense lesion, predominantly at the left posterior nasopharyngeal wall, measuring 8.2mm x 6.2mm, without any extension towards bilateral FOR (Figure 2a, 2b). 

**Fig 2a:**
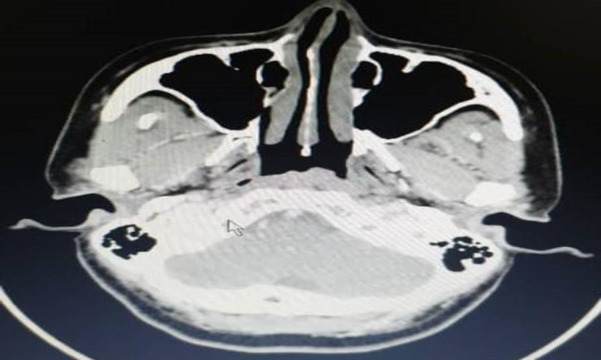
Fairly well-defined minimally enhancing hypodense lesion at the left posterior nasopharyngeal wall, measuring 8.2mm x 6.2mm, without any extension towards bilateral FOR

**Fig 2b:**
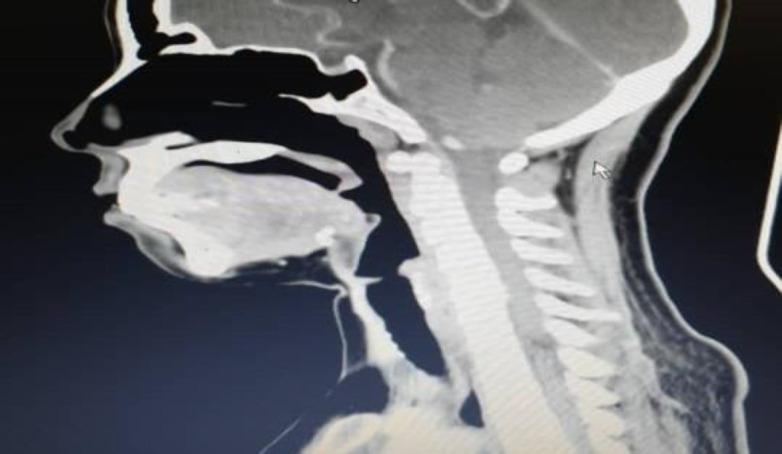
Sagittal view of CECT showed minimally enhancing lesion over the nasopharyngeal wall with irregular mucosa appearance

There were also enlarged rim-enhancing hypodense lesions over bilateral level 2 and left level 5a of the neck (Figure 2c). 

**Fig 2c:**
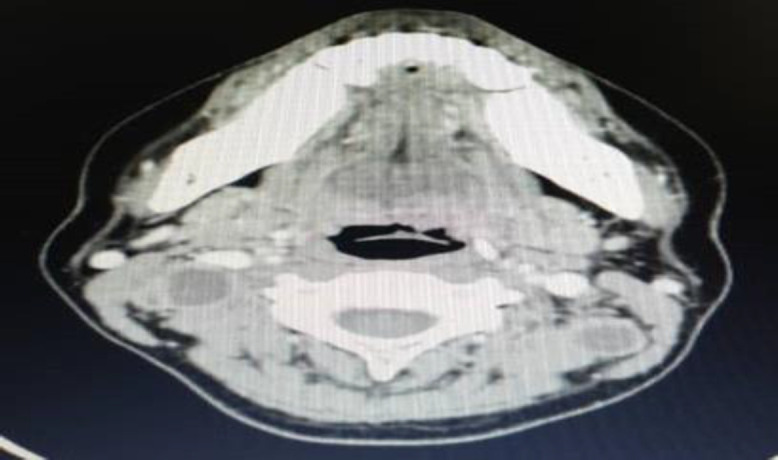
Enlarged rim enhancing hypodense lesions over bilateral level 2 and left level 5a of the neck

**Fig 3:**
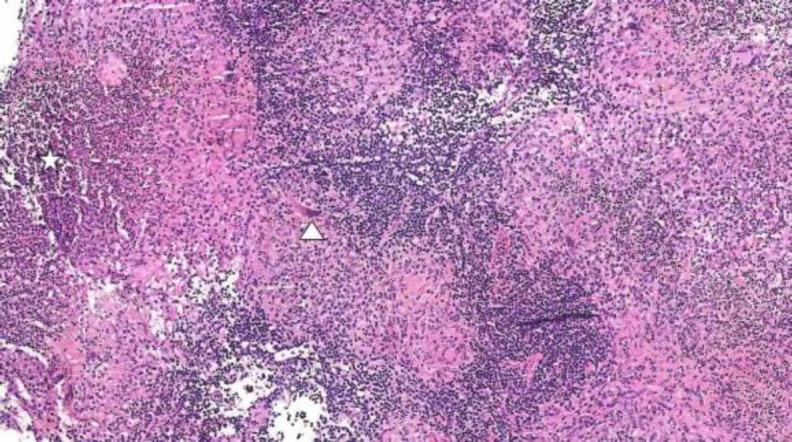
Granulomas with central areas of necrosis (star) surrounded by epithelioid cells mixed with lymphoplasmacytic cells and Langhans type multinucleated giant cells (arrowhead).- Haemat- oxylin and eosin stain, original magnification x 40

An excision biopsy of the left posterior triangle lymph node and a biopsy of the nasopharyngeal mass was performed under general anaesthesia. Histopathological examination (HPE) revealed epithelioid cell granulomas with central areas of necrosis surrounded by epithelioid cells mixed with lymphoplasmacytic cell infiltrates and occasional Langhans type multinucleated giant cells (Figure 3). 

No fungal elements, atypical or malignancy cells present. Surprisingly, the Ziehl-Neelsen (ZN) stain for acid-fast bacilli was negative. A further study performed on the paraffin-embedded tissue for mycobacterium (MTB) polymerase chain reaction (PCR) was also negative for acid-fast bacilli. Given the histopathological findings showing typical features of necrotizing granulomatous lymphadenitis together with the clinical presentation, a diagnosis of nasopharyngeal tuberculosis was made. She was referred to the chest clinic at Hospital Sultan Abdul Halim, Sungai Petani, for the commencement of directly observed treatment short-course (DOTS) for nasopharyngeal tuberculosis.

She was treated with an anti-tuberculous therapy regime consisting of Isoniazid, Rifampicin, Ethambutol, Pyrazinamide and Pyridoxine for 2 months during the intensive phase followed by 4 months of Isoniazid and Rifampicin during the maintenance phase. Upon completion of her intensive phase of treatment, her neck swelling had completely resolved, and the nasopharyngeal mass was no longer visualized.

## Discussion

Tuberculosis (TB) is a communicable disease that remained one of the leading causes of mortality worldwide. It can manifest particularly in either lung (pulmonary) or other sites (extrapulmonary). About 10-20% of immunocompetent and more than 40% of HIV-positive individuals were affected by extrapulmonary TB. Head and neck TB account for about 10% of extrapulmonary TB and the most commonly involved are the cervical lymph nodes(2). Primary nasopharyngeal tuberculosis (NPTB) is a rare disease even in an endemic area of tuberculosis, with or without pulmonary involvement. NPTB comprised only less than 1% of the head and neck TB(2). NPTB tends to coexist with pulmonary TB in about 1.9% of patients. Large series studies found that the mean age affected was between 30 and 40 years old with a slight predominance in women(3,4). Higher prevalence was found in those who are smokers, HIV-infected individuals and low socioeconomic status. Unfortunately, such findings were not pertinent to our case since she works as a teacher and her HIV screening was non-reactive.

Both primary NPTB in which an isolated nasopharyngeal tuberculous infection occurred without any other focus of tuberculous infection as evidenced in our patient and secondary NPTB by dissemination via haematogenous or lymphatic system were described for NPTB(4). The nasopharyngeal area, being an area of impact by inhaled air is easily overwhelmed by tuberculous droplets which then results in primary nasopharyngeal infection(3,5). The contaminated nasopharyngeal will expose the rich lymphatic networks of Waldeyer’s ring(4). Cervical lymphadenopathy with evidence of abnormal nasopharyngeal findings is the most common presentation followed by nasal discharge, nasal blockage, epistaxis and chronic cough. Other symptoms that had been reported include unilateral hearing loss, isolated 6^th^ cranial nerve palsy and sleep apnea(6). In addition to the usual presenting complaints, constitutional symptoms also can be found in 12-30% of NPTB cases (5). In our case report, she only presented with cervical lymphadenopathy and intermittent fever. Furthermore, it was supported by evidence of nasopharyngeal mass visualized via the nasoendoscopy examination. No atypical or other constitutional symptoms were demonstrated. According to Somchaiet et al, commonly encountered multiple bilateral lymphadenopathies were within the anterior neck triangle as compared to the posterior neck triangle. Retropharyngeal lymph nodes, which can only be visualized by imaging are commonly the first lymph node affected(3). Large 2 series studies reported that nasoendoscopy findings frequently revealed either irregular mucosa, normal mucosa or nasopharyngeal mass, which can often lead to a misdiagnosis. Evidence of polypoidal mass in the nasopharynx without surrounding soft tissue extension should raise a high index of suspicion for NPTB(4,5). Overlapping features and clinical presentation between NPTB, nasopharyngeal carcinoma and other malignant or infectious diseases may pose a great diagnostic challenge in which a misdiagnosis or suboptimal management will occur and resulting in a delay in the treatment, therefore altering the prognosis of the disease.

Numerous differential diagnoses should be considered when dealing with nasopharyngeal mass. Adenoid tissue, which usually disappears by the age of 16 years old, may be persistent in adults and mimic the appearance of a nasopharyngeal mass(7). Its persistence was thought to be related to allergy, chronic inflammation, infections, irritants or smoking. Meanwhile, nasopharyngeal carcinoma (NPC), lymphoma, plasmacytoma, rhabdomyosarcoma and chordoma are amongst malignancies that must be ruled out as delay in diagnosing is associated with significant morbidity and mortality(8,9). Benign cysts such as tornwaldt cyst, mucus retention cyst and bronchogenic cyst can be differentiated by their common location in the nasopharynx either midline or lateral, and are often found incidentally by nasoendoscopy or imaging. Other differentials include sarcoidosis, syphilis, Wegener granulomatosis and fungal infection. Nevertheless, to attain the correct diagnosis, nasoendoscopy findings alone which are not reliable to differentiate between benign and malignant lesions necessitates the use of histopathological and microbiological tests.

Computed tomography (CT) and magnetic resonance imaging (MRI) scan are helpful tools in illustrating the sites, pattern and disease extension, as some radiological characteristics might help in distinguishing between NPTB, malignancies and other differentials. Common radiological findings include polypoidal mass and mucosal thickening in the nasopharynx without extension to the prevertebral muscle, skull base, nasal cavity and oropharyngeal area. Evidence of small caseous necrosis within the nasopharyngeal lesion is one of the valuable hints towards NPTB(3). However, a similar sign might be present in the case of a large NPC tumour. Detection of Mycobacterium spp. bacterial culture from the tissue sample remained a gold standard for the diagnosis of NPTB(2). The bacterial culture and sensitivity test also can provide information regarding specific drug sensitivity towards the causative organism. To increase the chance of getting a positive bacterial culture, a tissue sample should be sent in the form of either a fresh sample or a small amount of normal saline as bacteria will be destroyed in a formalin-stained tissue sample (10). However, an average waiting time of four to six weeks might cause a delay in the diagnosis and treatment commencement. Typical histopathological findings of caseating granulomatous inflammation with multinucleated giant cells of Langhans type(11) combined with positive Ziehl-Neelsen staining for acid-fast bacilli were suggested to be helpful in the diagnosis of TB. Many authors suggested that the Ziehl-Neelsen staining is faster, easy to perform and inexpensive compared to the bacterial culture or PCR analysis. However, it requires intact and at least 10^4 ^bacilli per slide to be present and it has a low sensitivity which ranged only from zero to 44% (12). 

PCR analysis is usually reserved in highly suspicious cases of TB but with a negative for Ziehl-Neelsen stain and a negative bacterial culture. In comparison to Ziehl-Neelsen staining, PCR sensitivity was much higher ranging from 75% to more than 95% with a specificity of more than 95% in smear-positive TB. However, its sensitivity reduces to less than 90% in smear-negative TB(13). 

As shown in our case, possibly low sensitivity of Ziehl-Neelsen staining led to negative staining and negative bacterial culture for TB as a formalin-stained tissue sample was used for culture. Thus, typical histopathological findings confirmed the diagnosis even though not supported by other routine investigations.

Anti-tuberculous regime with 2 months’ intensive phase of Isoniazid, Rifampicin, Ethambutol and Pyrazinamide and 4 months maintenance phase of Isoniazid and Rifampicin were shown effective against both NPTB and pulmonary TB(4). 

NPTB carries a good prognosis and complete resolution is expected with adequate and optimal medical treatment. Fortunately, no cases of anti-tuberculous drug resistance or treatment failures were reported. As demonstrated in our case, the complete resolution of symptoms as well as nasoendoscopy findings was achieved after the intensive phase of anti-tuberculous treatment.

## Conclusion

 Nasopharyngeal tuberculosis is an uncommon disease with a great diagnostic challenge as many differential diagnoses need to be considered, particularly NPC, lymphoma and other malignancies. In addition, a wide range of clinical manifestations and less disease awareness might lead to the possible delay in the diagnosis and treatment. The presence of cervical lymphadenopathy with or without other associated symptoms should be followed by thorough ENT assessment, examinations and confirmation by histopathology and bacterial culture if needed. With early diagnosis and adequate treatment, NPTB carries a good prognosis with complete disease resolution.
